# Supplementary material for: Beyond social prescribing—The use of social return on investment (SROI) analysis in integrated health and social care interventions in England and Wales: A protocol for a systematic review
Source: PLoS One. 2023 Feb 21;18(2):e0277386. doi: 10.1371/journal.pone.0277386 (PMC9942995; doi:10.1371/journal.pone.0277386)
Supplement: S2 File — (PDF) [file pone.0277386.s003.pdf]

STUDENT AGREEMENT BETWEEN:

BANGOR UNIVERSITY

And

Mrs Genevieve Hopkins

STUDENT AGREEMENT

THIS AGREEMENT is dated the ………… day of …………………………………… 2021

BETWEEN:

(1) **BANGOR UNIVERSITY** (Registered Charity no. 1141565) whose office is at College Road, Bangor, Gwynedd, LL57 2DG (hereinafter referred to as the "University"); and,

(2) Genevieve Hopkins, whose address is Bryn Yr Eryr, Clynnog Fawr,, Caernarfon, Gwynedd, LL54 5PS (hereinafter referred to as the "Student").

RECITALS:

1) The University has successfully applied for funding from the European Social Fund Programme for East Wales, which is managed by the Welsh European Funding Office (hereinafter referred to as "WEFO") a part of the Welsh Government and the designated programme managing authority and certifying authority to deliver the "Knowledge Economy Skills Scholarship II (KESS 2) East Wales" operation (hereinafter referred to as "the Operation"). The Operation will support studentships leading to a Degree of Doctor of Philosophy (PhD) from Bangor University and support from the European Social Fund has been awarded to the University in WEFO's grant announcement letter dated **15th January 2019.**

2) The Operation will be focused on increasing the supply of individuals undertaking research and innovation activities at ISCED levels 7-8 in collaboration with, and driven by the needs of, the private sector, and focused on the Grand Challenge areas. The Operation will offer progression routes for individuals able to pursue qualifications at this level and responds to identified skills needs required to underpin economic growth and investment. KESS 2 East aims to contribute to the Welsh Government's aim to embed research and innovation in enterprises in the East Wales area by delivering demand led activities that align the development of higher-level skills with business benefits. Output indicators include employed participants, participants with graduate degrees (male/female), number of enterprises collaborating with learning providers, employed participants and female participants.

3) The terms under which the Student will participate in the Project are set out in this Agreement.

OPERATIVE PROVISIONS

1 The Project

1.1 The Student will undertake research work in the field of "Exploring the public's value of access to local leisure facilities" (the "Project") at the University under the supervision of Dr Mary Lynch, Dr Ned Hartfiel and Dr Elizabeth Mason (the "University Supervisor") of the School of Health Sciences. The University will notify the Student in writing if there is a change in supervisor.

1.2 The Student agrees to work in collaboration with Community Wellness Company (hereinafter referred to as "the Company") whose registered office is at Fernbank, Vicarage Lane, Gresford, Wrexham, LL12 8UW. The Student will undertake research at the Company's premises under the supervision of Karen Sankey (the "Company Supervisor") for one month per year during the Project period. The Company has agreed to notify the University in writing if there is a change in the Company Supervisor.

1.3 The Student will undertake a research project that has been agreed with the Company. The initial specification of the Project Application is as outlined in Appendix 1 of this Agreement. The University Supervisor in consultation with the Company and the Pan Wales KESS 2 Project Manager (or delegated authority) of the KESS Office, at the University must agree any significant variation from this initial specification in writing. Project Applications that are significantly changed will need to be approved by WEFO before the changes are applied.

1.4 The Student agrees to participate in the Project Postgraduate Skills Development Award ("PSDA") and agrees to participate in additional courses as directed by the Company Supervisor or the University Supervisor.

1.5 The Student agrees to complete monthly timesheets, which shall indicate an average of 39 hours of activity per week, and must be verified by the Company Supervisor or the University Supervisor as appropriate to the work undertaken and to submit verified timesheets to the University KESS Office REO-Finance on a Monthly basis. The Student may be required to repay any funding paid and the relevant proportion of tuition fees for periods where verified time sheets have not been provided. The University reserves the right to require the Student to keep timesheets under this clause 1.5 for the duration of the funding for auditing or other compliance reasons.

1.6 Subject to clause 7.1, the Student agrees to notify the University Supervisor in writing no less than 28 days in advance if they intend to leave the Project**.**

1.7 The Student can undertake up to 6 hours per week of teaching or demonstrating work, which is compatible with the Student's research Project. Other forms of work directly related to the Student's training will be allowed on the same conditions as for teaching/demonstrating. The Student may also undertake a small (6 hours maximum) amount of other paid work provided the Company Supervisor and the University Supervisor confirms their agreement, and the student confirms in writing that the work will not delay or otherwise interfere with the research training for which the studentship has been awarded.

1.8 If the Student is absent for medical reasons the Company Supervisor together with the University Supervisor will establish whether the absence will prevent the Student from completing the Project satisfactorily. The funding cannot be paid during extended periods of sickness and a decision will be made as to whether the funding should be withdrawn in such a case. If the Student is absent from the University for a duration which, in the opinion of the University Supervisor is unacceptable to the University Supervisor for reasons (other than medical reasons) the Student will normally forfeit the funding (including the waived tuition fees).

1.9 The Student Annual Leave Entitlement is 27 working days with the choice of dates left to the individual, subject to approval of the University Supervisor and Company Supervisor, who will not unreasonably withhold consent. The normal holiday's year runs from the 1st August to the 31st July. There are normally an additional 8 public holidays plus customary holidays. (http://www.bangor.ac.uk/humanresources/holidayshours.php.en)

1.10 The University must share certain information with third parties as detailed in appendix 3 of this Agreement. By signature of this Agreement, the Student consents to such information being shared.

1.11 It is a funding requirement that the Student must adhere to the ESF eligibility criteria throughout the entire project period in order that they remain eligible to receive the funding.

2 Duration

2.1 The Project will commence on the 18th January 2021 and will continue for a period of not more than three years, expiring on the 31st December 2023.

2.2 Notwithstanding clause 2.1, the obligations of the parties under this Agreement shall continue for the further duration of the three years.

3. Payment

3.1 The Project provides a stipend of £14,628 for the year 1 to be paid monthly in arrears by BACS payment on receipt of verified monthly timesheets and to the bank account as detailed at appendix 4. The stipend will increase to £14,774 in year 2 and to £14,348 in year 3. The first payment under this Agreement will be paid in arrears at the end of the first month after commencement of the Project and will be subject to the completion of a timesheet as required by clause 1.5. The Student must formally register with the Academic Registry of the University at the start of the Project and confirm continued attendance as required by the University for the Stipend Payments to continue. Failure to register by the date notified by the University will result in the stipend payments being withheld.

3.2 The University will waive the EU/UK tuition fees for the duration of the Project, subject always to the Student's adherence to the terms of this Agreement. Funding is also available to cover reasonable travel costs and attendance at conferences agreed and approved by the University Supervisor. These payments are subject to audit and the Student must therefore ensure that appropriate expense claims together with supporting documentation (including but not limited to original receipts and tickets for fares) is forwarded to the University Supervisor for final approval and processing.

3.3 The University and the Student agree that the Student is able to benefit from the provision available. The funding is provided on the basis of regular attendance at the University and satisfactory performance. The University Supervisor will decide what constitutes regular attendance and this decision will be final. The Student will keep detailed records of work undertaken.

3.4 The Student shall adhere to all Bangor University rules and regulations (https://www.bangor.ac.uk/regulations/regulations/reg13.php.en). However, where those rules and regulations conflict with this agreement, the terms of this agreement shall prevail.

3.5 The University may vary the conditions of this funding from time to time as necessary to meet the terms and conditions of the European Social Fund. Recipients of ESF funding will be expected to comply with such variations as notified by the University from time to time.

3.6 This Agreement is offered following completion of an ESF Participant Proposal form (the "Participant Record") and the eligibility evidence provided by the Student and based on information therein the University has determined that the Student is eligible to receive an award of funding supported by the European Social Fund. In the event that any of the information submitted by the Student in the Participant Record is later found to be untrue or misleading, the Student may be liable to repay all such funding received under this Agreement.

3.7 Students must abide by the rules in the University's Health and Safety Policies and Student Handbooks https://www.bangor.ac.uk/hss/students/documents/Student%20Handbook2020.pdf . Students studying away from the University must abide by the Health and Safety procedures of the Company. Actions that endanger the Health and Safety of others may lead to disciplinary action by the University and to prosecution under the Health and Safety at Work Act, 1974.

4. Publicity and Confidentiality

4.1 As a condition of participating in the Operation and the Project, the Student agrees to keep all confidential, secret or proprietary information of the University and/or the Company as is disclosed under this Agreement, or which the Student receives or generates including Project Intellectual Property Results ("IPR") (the "Confidential Information"), secret and confidential and agrees not to disclose, divulge or communicate it to anyone else, except to another person who is involved in the Project and has given an equivalent undertaking of confidentiality.

4.2 The obligations contained in this clause 4 shall survive the expiry or termination of this Agreement for any reason but shall not apply to any Confidential Information which:

4.2.1 is known to the party making the disclosure before its receipt from the other party, and not already subject to any obligation of confidentiality to the other party;

4.2.2 is or becomes publicly known without any breach of this Agreement or any other undertaking to keep it confidential;

4.2.3 has been obtained by the party making the disclosure from a third party in circumstances where the party making the disclosure has no reason to believe that there has been a breach of an obligation of confidentiality owed to the other party;

4.2.4 has been independently developed by the party making the disclosure;

4.2.5 is disclosed pursuant to the requirement of any law or regulation or the order of any Court of competent jurisdiction, and the party required to make that disclosure has informed

the other, within a reasonable time after being required to make the disclosure, of the requirement to make the disclosure and the information required to be disclosed.

4.3 The Student will not publish any material arising from the Project without prior written approval of the University. Such approval will not be unreasonably withheld but may be subject to delay pending assessment of the proposed publication. The University will have 45 days, after receipt of said copies to object to such proposed publication, because there is patentable or commercially sensitive subject matter, which needs protection or because the Company has requested deletion of its Confidential Information. In that event, the Student will refrain from making such publication for a maximum of twelve months from date of receipt of such objection in order for a patent application or other protection to be filed. If no written reply is received within 45 days of receipt of the document, permission for publication will be deemed to have been given.

4.4.1 The Student and the University will ensure that all publicity documents acknowledge the funding received from the European Social Fund in accordance with the information supplied in Appendix 2.

4.4 The publication of results arising from the Project should acknowledge the support of the European Social Fund and the Company.

4.5 Within 6 months of the end of the duration of the Project, a thesis must be submitted for examination. This requirement shall prevail over any of the University's policies relating to submission of theses.

5. Reporting

5.1 The Student will maintain regular contact with the Company, and will co-operate as required with the University to submit interim progress reports to the Company setting out recent developments of any research carried out under the Project.

5.2 The Student will co-operate with the University in order to submit to the Company a copy of a final report within 90 days of completion of the Project.

5.3 The Student will maintain any findings, the interim and final reports in strict confidence and will not divulge the contents thereof to any outside party other than in accordance with Clause 4.

6. Proprietary Rights

6.1 All background intellectual property rights belonging to one Party is and shall remain the exclusive property of the Party owning it (or where applicable, the third party from whom it's right to use the background intellectual property has derived).

6.2 Each Party grants to the other Party a royalty-free, non-transferrable, non-exclusive, license to use its background intellectual property for the sole purpose of the performance of the Project.

6.3 All Intellectual Property Rights created or invented by the Student in the course of undertaking work on the project ("IPRs") shall be owned by the University.

6.4 In consideration of the University offering the Student the KESS 2 Scholarship funding the Student hereby assigns to the University absolutely with Full Title Guarantee, all of their right, title and interest in the following rights: (a) all IPR (created or invented or are in the future); and (b) the right to sue for damages and other remedies for infringement of any of the IPR. ("Full Title Guarantee" to be defined as that which the Student: is entitled to sell; will do all that is necessary, at his/her own expense, to vest title to the property in the University; and, shall ensure that the property is free from encumbrances and adverse rights other than those which the Student does not and could not reasonably know about").

6.5 The Student also agrees to waive their moral rights as far as they can.

6.6 The Student shall provide a written list of any IPR which the Student already owns which may be used in connection with the Project before the Project starts.

6.7 To give effect to 6.4 and 6.5, the Student also agrees to do all things and sign all documents that the University reasonably requires confirming the University's ownership of the IPRs. This will be at the University's reasonable cost and expense. The University will try to ensure that the Student is given attribution for the work that the Student carries out under the Project and that the Student receives an equitable share of any benefits arising from the IPRs (which

could include a share of any royalties) in accordance with the University's Intellectual Property Policy as amended from time to time.

6.8 If any of the IPRs which are assigned to the University have not been exploited by the University (in the University's opinion) within five (5) years of the Student ceasing to be a student at the University, the University will reassign such IPRs that have not been exploited in any way to the Student if the Student so requests in writing. The Student shall reimburse the University for all reasonable costs incurred by it for any assignment of the IPRs to the Student.

7. Failure to complete

7.1 If the Student withdraws from the Project or fails to complete the Project (including but not limited to the submission of the theses within 6 months of the Project end date and the achievement of the 60 credit Postgraduate Skills Development Award) for any reason they may be liable to repay the University the funding received under clause 3 of this Agreement.

8. Termination

8.1 The University may terminate this Agreement forthwith if the Student:-

8.1.1 Is in breach of this Agreement and fails to remedy such breach (where such breach is capable of remedy) within 30 days of the receipt of a request in writing from the University to remedy the breach; or,

8.1.2 Is in breach of any of the confidentiality provisions in clause 4.

8.2 The University may terminate this Agreement in the event that the Company ceases to contribute to the Project or in the event that the funding which the University receives in relation to the Project is ceased.

8.3 The University may terminate the Student's registration and/or this Agreement in the following circumstances:

8.3.1 there is lack of academic progress;
8.3.2 there is a loss of contact;
8.3.3 the Student refuses to assign any IPR upon request; and/or
8.3.4 the student's behavior is deemed to be unsatisfactory and the Student is required to withdraw their registration for their programme of study at the conclusion of the University's Disciplinary Procedures. In all cases, the Student Disciplinary Procedures will be followed before termination of the Student's registration. The Student shall have the right of appeal in respect of any decision to withdraw them.

8.4 This Agreement shall terminate upon termination of the Student's registration at the University.

8.5 In the event of expiry or termination of this Agreement howsoever arising clauses 4, 6, 7.1, 9.2 and 11 will survive.

9. Monitoring requirements

9.1 The Student agrees to notify the University of their Employment Destination at the end of their funded period, 6 months and one year after the Project period. This requirement will apply even if the Student is unemployed or is looking for work.

9.2 The Student may be contacted at any time during the 3 years period following the end of the Project and agrees to respond promptly to requests for information.

10. Third Party Rights

10.1 The parties to this Agreement do not intend that any of its terms will be enforceable by virtue of the Contracts (Rights of Third Parties) Act 1999 by any person not a party to it, other than by the Company for any breaches of confidentiality by the Student in accordance with clause 4.

11. Governing Law

11.1 This Agreement will be governed by the laws of England and Wales and the parties agree to submit to the exclusive jurisdiction of the Courts of England and Wales

12. General

12.1 This Agreement, together with any documents referred to in it, constitutes the whole agreement and understanding between the parties and supersedes any previous agreement between the parties relating to the subject matter of this Agreement.

12.2 If any provision of this Agreement will be found by any court or administrative body of competent jurisdiction to be invalid or unenforceable, the invalidity or unenforceability of such provision will not affect the other provisions of this Agreement and all provisions not affected by such invalidity or unenforceability will remain in full force and effect.

12.3 The waiver by either party of a breach or default of any of the provisions of this Agreement by the other party will not be construed as a waiver of any succeeding breach of the same or other provisions as save as expressly stated herein, neither will any delay or omission on the part of either party to exercise or avail itself of any right, power or privilege that has or may have hereunder operate as a waiver of any breach or default by the other party.

12.4 This Agreement may only be amended by a document in writing signed by a duly authorised signatory of both parties.

13. Environmental Information Regulations & Freedom of Information

13.1 The Student acknowledges that the University is subject to requirements under the Environmental Information Regulations 2004 (“EIR”) & Freedom of Information Act 2000 (“FOIA”) and shall assist and cooperate with the University to enable the University to comply with any information disclosure requirements.

13.2 Where the University receives a request to disclose any information that, under this Agreement, is the Student’s Confidential Information, it will notify the Student and will consult with the Student. The Student shall respond to the University within five (5) days after receiving the University’s notice of the request. In the event that the Student fails to respond within the requisite period, the University reserves the right to disclose any such information it deems appropriate.

13.3 The University shall be responsible for determining at its absolute discretion whether the information is:-

13.3.1 exempt from disclosure in accordance with the EIR & FOIA;

13.3.2 to be disclosed in response to a request for information under the EIR & FOIA and in no event shall the Student respond directly to a request for information unless expressly authorised to do so by the University;

13.4 The Student acknowledges that the University may be obliged under the EIR & FOIA to disclose information following consultation with the Student and having taken its views into account.

IN WITNESS WHEREOF the parties or their duly authorised representative or representatives the day and year first above written

Signed (Student): Genevieve Hopkins ....

Name (Block Capitals): Genevieve Hopkins ....

Position: PhD Student

Date: 25/01/2021 ....

Signed (on behalf of the University): ………………………………………

Name (Block Capitals): BEN DAVIES

Position: Project Accountant - Structural Funds Manager

Date: ....

Appendix 1

Project Application

Appendix 2

Publicity Requirements

Appendix 3

INFORMATION ON THE USE OF PERSONAL DATA IN PROJECTS PART-FUNDED FROM THE STRUCTURAL FUNDS 2014 TO 2020.

Bangor University is responsible for managing the KESS 2 operation on behalf of the HE sector in Wales. KESS 2 is part-funded by the Welsh Government's European Social Fund (ESF) through the East Wales Programme. The Welsh European Funding Office (WEFO), part of the Welsh Government, is the managing and Certifying Authority for the Structural Funds in Wales.

Personal Information

Bangor University is required to collect and use personal information supplied by you, to include your name, address, date of birth, level of education, employment status, telephone number, e-mail address, preferred language, date of birth, National Insurance Number, Employment status prior to registration, Education, Career, employment, bank / building society statements, passport, driving licence, NI number, Identity cards, correspondence from UK Immigration, birth / adoption certificate, marriage / civil partnership certificate – if partner has legal right to live in the UK, Qualifications, employment contract, payslips, evidence confirming receipt of age-related state benefits, Tenancy agreements, mortgage statement, utility bill, firearms certificates, shotgun licence, rent card and any alternative information that may be required; for the purpose of establishing your eligibility to receive support from the ESF. We need to be able to provide suitable justification of an assessment of your fulfilment of the entry conditions for receipt of ESF support. We may not be able to support you if you decide not to provide us with this information.

Details of your skills, qualifications, aspirations, interests, training needs and progress will also be gathered and retained. KESS 2 will require you to use an interactive website called Skills Forge to help you to manage your degree as well as managing the development of your personal and professional skills.

Special Category (Sensitive) Information

It is a condition of the registration of students that individuals agree to Bangor University's processing of specified classes of personal data, including special category (sensitive) data. Some data, for example, those related to disability or ethnic status may be considered sensitive. This data is gathered as it helps the KESS 2 project to check that our recruitment practices are effective in encouraging all individuals to apply to participate in the project. It also helps us to provide additional support to students where required. The data will also help us to confirm that we comply with equality legislation.

Use of personal information

In addition to using the information to establish your eligibility and to help you with your progression through the project, we may use the information to:

- Contact you about additional opportunities that may become available
- Contact you to discuss your progress on the project

- Work with you to develop case studies and other marketing material to confirm the success of the project
- Support our internal record keeping
- Contact you to monitor and/or evaluate the success of the KESS 2 project
- Contact you to confirm your employment status when you have completed your participation in KESS 2

Storing the information.

The information given to the University will be kept strictly confidential and will only be shared with relevant staff within the University on a need to know basis for the purpose of project administration.

Whilst you are taking part in KESS 2 the University will keep your details on file. Once you have completed the project it is a condition of the award that the information is kept by the University for at least ten years after the end of the KESS 2 project, although this may be subject to change and will be guided by WEFO retention requirements, but rest assured the information is still kept securely and confidentially throughout the archival period.

Sharing personal information.

It is a requirement of the Structural Funds Programmes that the University is required to send the information it holds on you to the WEFO for the purpose of monitoring and evaluating projects part-funded through the Structural Funds Programmes in Wales. The WEFO will pass on this personal data to a third party contractor acting on their behalf for the purposes of research, evaluation and verification of financial support provided from the Structural Funds. Your personal data may also be passed to a third party organisation that the University appoints to carry out an evaluation of the KESS 2 operation. Data that is provided to WEFO will be processed in accordance with the requirements of the Data Protection Act 2018. The Welsh Ministers are registered as a data controller on the Information Commissioner's public register of data controllers under registration number Z7107446. Personal data will not, without consent, be made public in any way that identifies individuals. Certain data will be passed to the European Commission in compliance with Structural Funds Regulations.

In line with Article 6 and 7 of the Directive 95/46/EC of 24 October 1995 on the protection of individuals with regard to the processing of personal data and on the free movement of such data, the ESF Regulation provides the legal basis to justify collection and processing of personal data for the purposes of monitoring and reporting on ESF funded actions.

If you would like to request details of your personal data being held on the WEFO central database you may contact their Helpline on 0845 010 3355.

Copy of information

You should be aware that under the terms of the Data Protection Act 2018, you have the right to a copy of the information which the University holds about you. The University's procedure for accessing information can be found here:- https://www.bangor.ac.uk/governance-and-compliance/dataprotection/DPRequest.php.en

Alternatively, you are invited to contact the member of staff responsible for data protection compliance at the University:-

Gwenan Hine, Head of Governance and Compliance, Governance and Compliance Office, Bangor University, College Road, Bangor, Gwynedd LL57 2DG e-mail: info-compliance@bangor.ac.uk
